# Thrombomodulin promotes focal adhesion kinase activation and contributes to angiogenesis by binding to fibronectin

**DOI:** 10.18632/oncotarget.11828

**Published:** 2016-09-02

**Authors:** Yun-Yan Hsu, Guey-Yueh Shi, Kuan-Chieh Wang, Chih-Yuan Ma, Tsung-Lin Cheng, Hua-Lin Wu

**Affiliations:** ^1^ Department of Biochemistry and Molecular Biology, College of Medicine, National Cheng Kung University, Tainan, Taiwan; ^2^ Cardiovascular Research Center, College of Medicine, National Cheng Kung University, Tainan, Taiwan; ^3^ Department of Physiology, College of Medicine, Kaohsiung Medical University, Kaohsiung, Taiwan; ^4^ Orthopaedic Research Center, College of Medicine, Kaohsiung Medical University, Kaohsiung, Taiwan

**Keywords:** endothelial cells, VEGF, thrombomodulin, fibronectin, angiogenesis

## Abstract

Angiogenesis promotes tumor growth and metastasis. Cell adhesion molecules interact with the extracellular matrix (ECM) and increase cell adhesion and migration during angiogenesis. Thrombomodulin (TM) is a cell surface transmembrane glycoprotein expressed in endothelial cells. However, the function and significance of TM in cell-matrix interactions and angiogenesis remain unclear. Here, we first demonstrated that recombinant lectin-like domain of TM interacts with an ECM protein, fibronectin, and identified the N-terminal 70-kDa domain of fibronectin as the TM-binding site. Exogenous expression of TM in TM-deficient A2058 melanoma cells enhanced cell adhesion and migration on fibronectin and invasion on Matrigel. In addition, TM increased focal adhesion kinase (FAK) phosphorylation and matrix metalloproteinase-9 production. In mice bearing subcutaneous B16F10 melanoma tumors, immunofluorescence analysis indicated that TM was highly expressed and co-localized with fibronectin on the tumor vasculature. The interaction between TM and fibronectin in tumor blood vessels was also validated by the proximity ligation assay. In human umbilical vein endothelial cells, up-regulation of TM by vascular endothelial growth factor (VEGF), a tumor angiogenic factor, promoted cell adhesion and tube formation, whereas TM knockdown by RNA interference attenuated VEGF-induced cell adhesion and tube formation. In summary, TM promotes angiogenesis by enhancing cell adhesion, migration, and FAK activation through interaction with fibronectin. TM may represent a novel target for inhibiting tumor angiogenesis.

## INTRODUCTION

Cell surface adhesion molecules interact with the extracellular matrix (ECM) to regulate cell adhesion and migration during embryonic development, organ morphogenesis, and angiogenesis [1, 2]. The ECM proteins, including vitronectin, fibronectin, laminin, and various collagens, not only serve as substrates for cell adhesion and migration but also provide a scaffold for tissue integrity [3]. Integrins can mediate cell-ECM interactions and regulate cell adhesion, migration, gene expression, and matrix remodeling [3, 4]. After binding to the ECM, integrins initiate signal transduction by recruiting various signaling and adaptor proteins via their cytoplasmic domains [3]. Focal adhesion kinase (FAK) plays a key role in integrin signaling and regulates cell migration [3, 5]. In response to integrin-mediated cell adhesion, FAK is phosphorylated at several tyrosine residues, including Tyr397, Tyr576, and Tyr577 [5]. Integrins promote FAK Tyr397 autophosphorylation, which creates a binding site for Src. Subsequently, phosphorylation of Tyr576 and Tyr577 by Src leads to maximal FAK activity [5]. In addition to integrins, other cell surface molecules, including syndecans and CD44, have been reported to mediate cell-ECM interactions [6, 7].

Thrombomodulin (TM) is a cell surface transmembrane glycoprotein that was originally identified as an anticoagulant factor on the vascular endothelium [8]. TM knockout leads to embryonic lethality in mice [9]. Structurally, TM consists of 5 domains, including an N-terminal lectin-like domain, an epidermal growth factor (EGF)-like domain containing 6 EGF-like structures, a serine/threonine-rich domain, a transmembrane domain, and a cytoplasmic domain [8]. TM is expressed in various cell types and exerts multiple biological functions via its different domains [8]. TM plays important roles in regulating blood coagulation, inflammation, and skin wound healing [8, 10]. Our earlier study showed that TM mediates cell-cell adhesion through its lectin-like domain [11]. Recently, we demonstrated that TM deficiency reduces macrophage adhesion to endothelial cells [12]. In addition, we found that the cytoplasmic domain of TM associates with ezrin, which links TM to the actin cytoskeleton [13]. However, the role of TM in cell-matrix adhesion has not been characterized.

We previously found that different recombinant domains of soluble TM positively or negatively regulate angiogenesis. The recombinant TM containing an EGF-like domain and a serine/threonine-rich domain enhances angiogenesis by promoting endothelial cell proliferation and migration [14], while the recombinant TM containing a lectin-like domain suppresses angiogenesis by interfering with EGF-mediated angiogenic effects via interacting with Lewis Y antigen of the EGF receptor [15]. To date, the function of membrane-bound TM in angiogenesis is poorly understood. Previous reports showed that interactions of endothelial integrins with ECM are needed for angiogenesis [2, 16]. TM is a cell surface-localized protein, but whether TM interacts with the ECM remains undetermined. Structurally, TM contains 5 domains as mentioned above. The domain structure of TM suggests that the lectin-like domain, but not the EGF-like domain, of TM, is farthest from the plasma membrane and close to the ECM. Therefore, we tested the interaction of the TM lectin-like domain with the ECM. Subsequently, we investigated whether cell surface TM, which is the complete TM molecule, plays a role in regulating cell-matrix interactions and angiogenesis.

In the present study, we investigated whether TM interacts with the ECM and contributes to angiogenesis. It remains unclear which of the TM domains has the potential to interact with the ECM. Based on the domain structure of TM, the TM lectin-like domain might be close to the ECM. We therefore analyzed the interaction of recombinant TM lectin-like domain (rTMD1) with various ECM proteins using the solid-phase binding assay. We identified fibronectin, which is a crucial ECM molecule involved in angiogenesis, as a novel ligand for TM. The possible roles of cell surface full-length TM in cell adhesion, migration, FAK activation, and angiogenesis were investigated. This study may help understand the significance of membrane-bound TM in tumor angiogenesis.

## RESULTS

### rTMD1 interacts mainly with fibronectin

**Figure 1:**
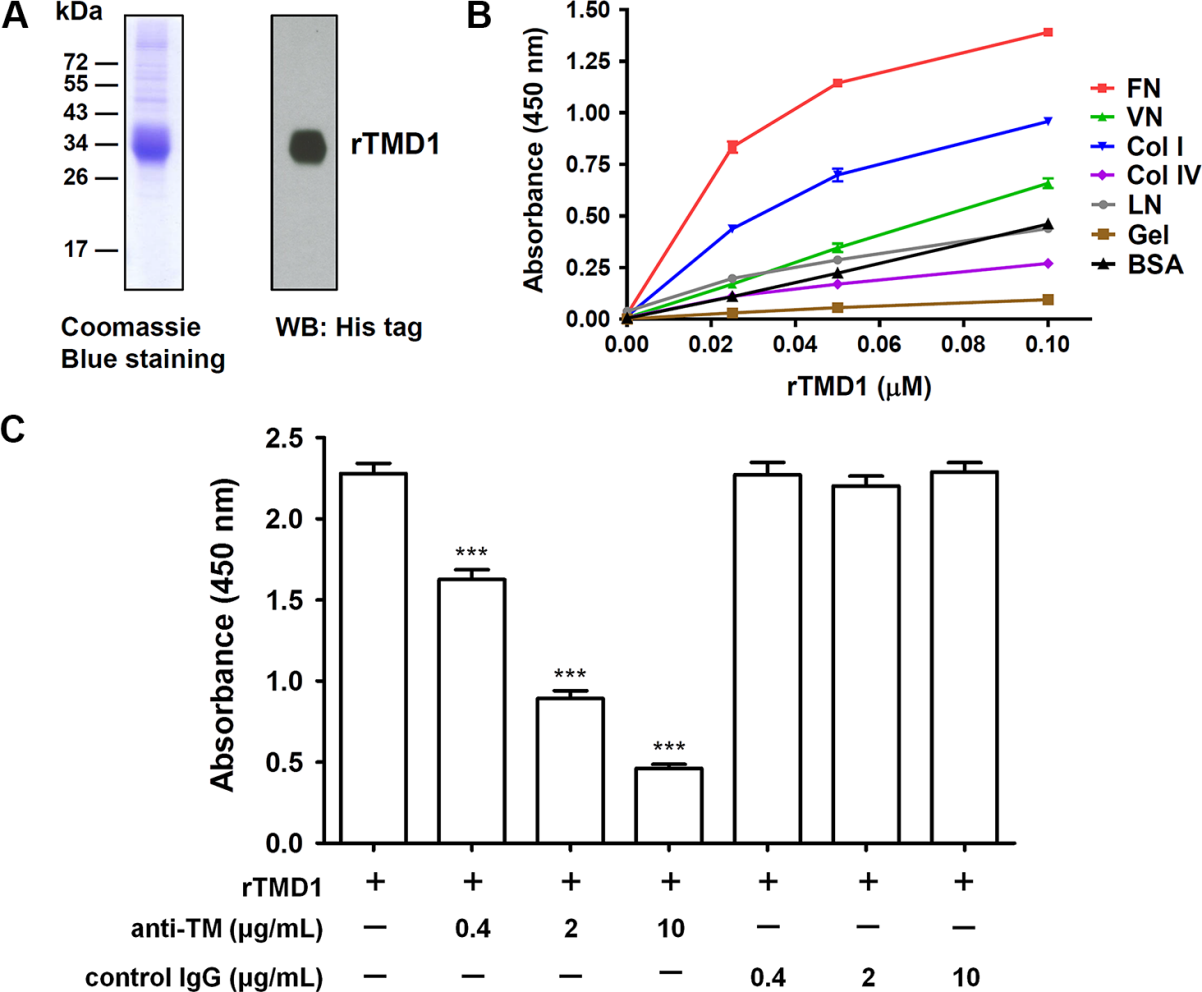
rTMD1 interacts specifically with fibronectin (**A**) Coomassie Blue staining and western blotting (WB) of purified rTMD1. (**B**) Binding of rTMD1 to ECM proteins. The wells of a Maxisorp plate were coated with 10 μg/mL of fibronectin (FN), vitronectin (VN), collagen I (Col I), collagen IV (Col IV), laminin (LN), gelatin (Gel), or BSA. The wells were incubated with increasing concentrations of rTMD1. Bound rTMD1 was detected using an anti-His antibody. Values are means ± SD of triplicate wells. (**C**) rTMD1 (0.1 μM) was incubated with or without increasing concentrations of the anti-TM antibody for 1 h and then added to fibronectin-coated wells. Nonimmune mouse IgG was used as a control. Bound rTMD1 was detected using an anti-His antibody. Values are means ± SD of triplicate wells. Data are representative of 3 independent experiments. ***P < 0.001 compared with rTMD1 alone.

To investigate whether the lectin-like domain of TM directly interacts with ECM proteins, we first performed solid-phase binding assays. rTMD1 (Ala^1^ through Ala^155^) with c-Myc and His tags was prepared with the mammalian protein expression system. The purified rTMD1 was homogeneous as judged by SDS-PAGE and western blotting (Figure 1A). Various ECM proteins, including collagen type I, type IV, fibronectin, laminin, vitronectin, and gelatin (denatured collagen), were commercially available and immobilized onto wells of the plate in the solid-phase binding assay. rTMD1 mainly interacted with fibronectin in a dose-dependent manner and bound weakly to collagen type I (Figure 1B). Using bovine serum albumin (BSA) as a control for nonspecific binding, rTMD1 did not bind to laminin, collagen type IV, and gelatin and bound very weakly to vitronectin (Figure 1B). We excluded the binding of the anti-His antibody to the ECM proteins since the anti-His antibody did not bind to the ECM proteins in the absence of rTMD1 (0 μM rTMD1 in Figure 1B). To verify the specificity of the interaction between rTMD1 and fibronectin, we performed an inhibition assay using a mouse monoclonal antibody against the TM N-terminal region. rTMD1 binding to fibronectin was inhibited in a dose-dependent manner by the anti-TM antibody, and the binding was not affected by the nonimmune mouse IgG (Figure 1C). These data indicate that rTMD1 binds directly and mainly to the ECM fibronectin. In addition, the binding of rTMD1 to fibronectin was the same when using phosphate-buffered saline (PBS, pH 7.4) instead of carbonate buffer as the coating buffer (data not shown).

### rTMD1 binds to the N-terminal 70-kDa domain of fibronectin

**Figure 2:**
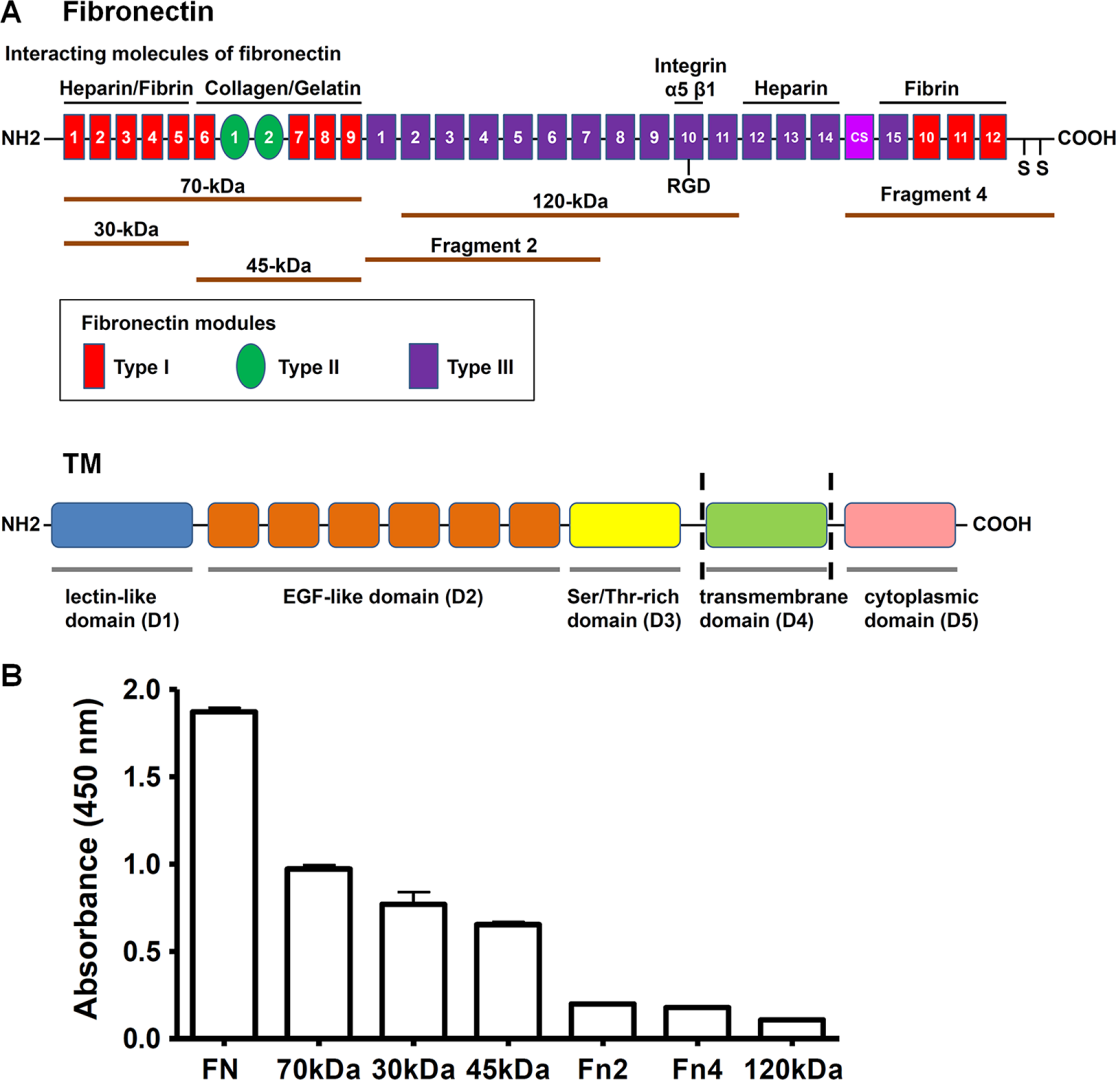
rTMD1 binds to the N-terminal 70-kDa domain of fibronectin (**A**) Top: A schematic diagram of a plasma fibronectin monomer shows ligand-binding sites and the fibronectin proteolytic and recombinant fragments used in this study. Bottom: A schematic diagram shows structural domains of TM. (**B**) rTMD1 binding to fibronectin and its proteolytic and recombinant fragments. Intact fibronectin (10 μg/mL) and equimolar amounts of various fibronectin fragments were coated onto wells. After blocking with 1% BSA, rTMD1 (0.1 μM) was added to wells. Bound rTMD1 was detected using an anti-c-Myc antibody. Values are means ± SD of triplicate wells. Results are representative of 3 independent experiments.

Fibronectin is a dimer composed of two similar 230–270 kDa monomers joined by two disulfide bonds at the C-terminus [17]. A fibronectin monomer contains three types of repeating modules, termed type I, type II, and type III. Fibronectin was reported to bind to a number of important molecules, including heparin, fibrin, collagen, gelatin, and integrins [1]. To identify the region of fibronectin involved in the interaction with rTMD1, we determined the interactions of rTMD1 with different fragments of fibronectin. The top of Figure 2A illustrates a monomer of plasma fibronectin and some of its ligand-interaction sites and shows the fibronectin proteolytic and recombinant fragments used in our study. The N-terminal 70-kDa fragment comprises the 30-kDa heparin/fibrin-binding domain and the adjacent 45-kDa collagen/gelatin-binding domain. The central 120-kDa fragment contains the type III2–11 modules with the Arg-Gly-Asp (RGD) motif in the type III10 module. Recombinant fibronectin fragment 2 contains the type III1–7 modules, and fragment 4 consists of the type III connecting segment (IIICS), one type III module, three type I modules, and the site of interchain disulfide linkage. The bottom of Figure 2A shows a schematic diagram of structural domains of TM. In addition to intact fibronectin, rTMD1 mainly interacted with the N-terminal 70-kDa fragment and its proteolytic cleavage fragments (30-kDa and 45-kDa fragments), but not the recombinant fibronectin fragment 2, fragment 4, or the central 120-kDa fragment (Figure 2B). On the other hand, the binding of rTMD1 to fibronectin was independent of the His and c-Myc tags because the binding could be detected by the anti-His and anti-c-Myc antibodies (Figures 1B, 1C, and 2B).

### Exogenous expression of TM enhances cell adhesion on fibronectin and increases FAK tyrosine phosphorylation

**Figure 3:**
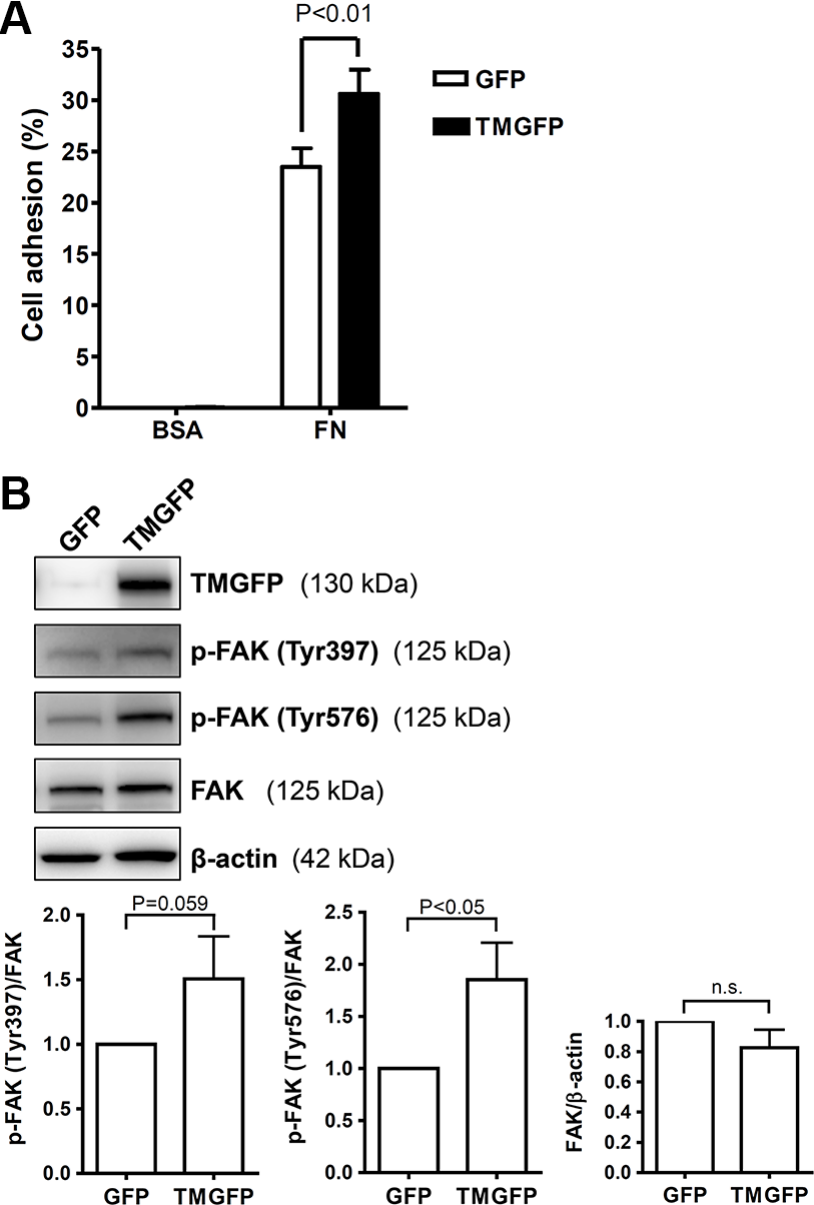
Exogenous expression of TM enhances cell adhesion on fibronectin and increases FAK tyrosine phosphorylation (**A**) A2058 cells expressing GFP or GFP-tagged TM (TMGFP) were loaded onto fibronectin-coated wells and incubated for 1 h at 37°C. After washing, adherent cells were quantitated by measuring endogenous cellular phosphatase activities as described in Materials and Methods. Data were expressed as the percentage of adherent cells to total cells loaded. Values represent means ± SD of quadruplicate wells. (**B**) A2058 cells expressing GFP or GFP-tagged TM were plated on fibronectin-coated dishes for 1 h at 37°C. Lysates of adherent cells were analyzed by western blotting with antibodies against TM, phospho-FAK (p-FAK) (Tyr397), phospho-FAK (Tyr576), FAK, or β-actin. Band intensities were quantified using ImageJ. Top panels are representative of 3 independent experiments. Bottom panels show quantitative results of phospho-FAK levels normalized by FAK and that of total FAK levels normalized by β-actin. Values represent means ± SD of 3 independent experiments. n.s., not significant.

Based on the result that the TM lectin-like domain binds predominantly to fibronectin, we further explored the effect of TM on cell adhesion to fibronectin. TM-deficient melanoma A2058 cells were transfected with plasmids encoding green fluorescent protein (GFP)-tagged TM or GFP control, and stable cell lines were used to compare the adhesion capability. GFP-tagged TM-expressing A2058 cells exhibited 1.3-fold increased adhesion on fibronectin compared with GFP-expressing cells (Figure 3A). In this assay, the increased cell adhesion upon exogenous TM expression is modest, possibly due to the endogenous expression of other fibronectin receptors such as integrins. In addition, we performed a cell adhesion assay using collagen IV as a substrate. The result showed that TM did not increase cell adhesion on collagen IV (Supplementary Figure S1). FAK is phosphorylated and activated following integrin-mediated cell-matrix interactions [5]. Given that TM enhanced cell adhesion on fibronectin, we next determined whether TM modulates FAK phosphorylation. A2058 cells expressing GFP or GFP-tagged TM were plated on fibronectin-coated dishes for 1 h, and lysates of adherent cells were analyzed by western blotting. The results showed that FAK phosphorylation levels on Tyr397 and Tyr576 were higher in GFP-tagged TM-expressing cells than in GFP-expressing cells (Figure 3B). The total FAK levels were not significantly different between GFP-expressing cells and GFP-tagged TM-expressing cells (Figure 3B). These results indicate that TM increases cell adhesion on fibronectin and FAK phosphorylation on Tyr397 and Tyr576.

### Exogenous expression of TM enhances cell migration and invasion

**Figure 4:**
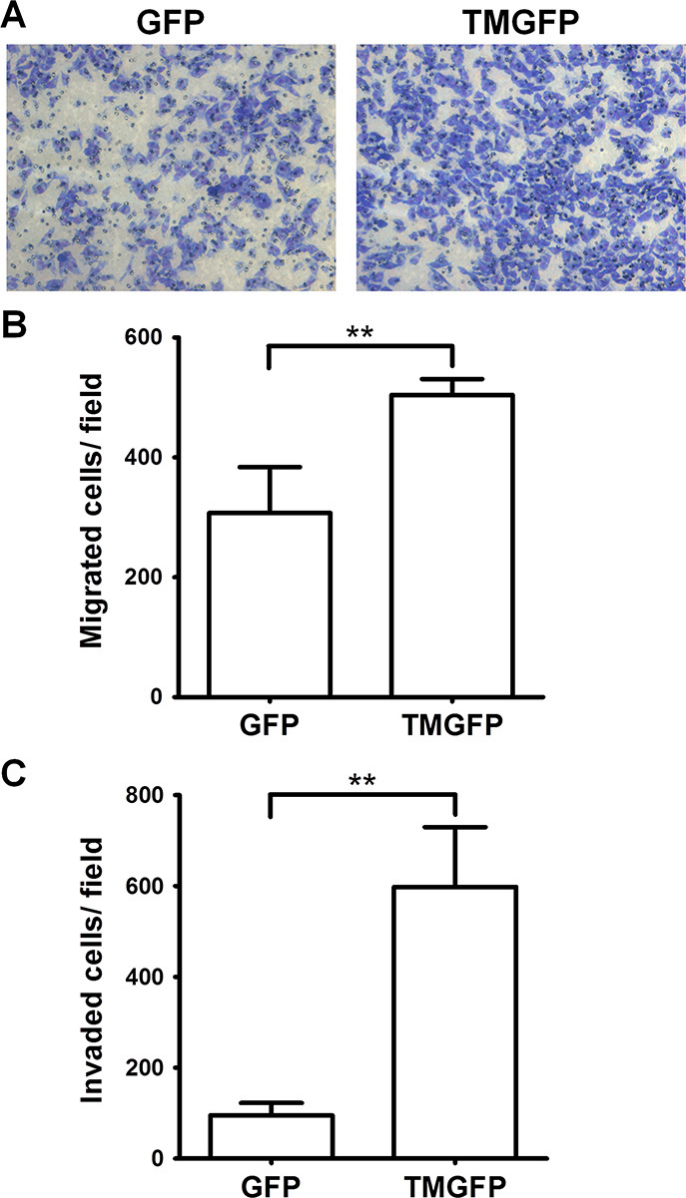
Exogenous expression of TM enhances cell migration and invasion (**A–B**) A2058 cells expressing GFP or GFP-tagged TM (TMGFP) were loaded into the upper compartment of the Boyden chamber with a fibronectin-coated filter, and allowed to migrate in response to 5% FBS. After 4 h, migrated cells on the filter lower surface were stained with Liu's stain. Five fields per well were photographed under a microscope (×100 magnification) (A), counted, and averaged (B). Values represent means ± SD of quadruplicate wells. Data are representative of 3 independent experiments. (**C**) A2058 cells expressing GFP or GFP-tagged TM were loaded into the Matrigel-coated Transwell, and allowed to invade through Matrigel for 20 h. Invaded cells on the membrane lower surface were stained with Liu's stain. Three fields per well were photographed under a microscope (×100 magnification), counted, and averaged. Values represent means ± SD of triplicate wells. Data are representative of at least 3 independent experiments. **P < 0.01.

To investigate whether TM regulates cell migration on fibronectin, we analyzed the migratory abilities of GFP-expressing and GFP-tagged TM-expressing A2058 cells using Boyden chamber assays. Cell suspensions were loaded onto the fibronectin-coated filter in the Boyden chamber and allowed to migrate toward 5% fetal bovine serum (FBS). After 4 h of incubation, the number of cells that had migrated to the undersurface of the filter was determined. Compared with control cells expressing GFP, A2058 cells expressing GFP-tagged TM showed a 1.64-fold increase in migration through the fibronectin-coated filter (Figure 4A and 4B). This result suggests that cell surface TM promotes cell migration on fibronectin. To determine the effect of TM on cell invasion, we performed Matrigel invasion assays. The result showed that exogenous expression of TM in A2058 cells promoted cell invasion through Matrigel (Figure 4C).

### Exogenous expression of TM enhances matrix metalloproteinase-9 (MMP-9) production

**Figure 5:**
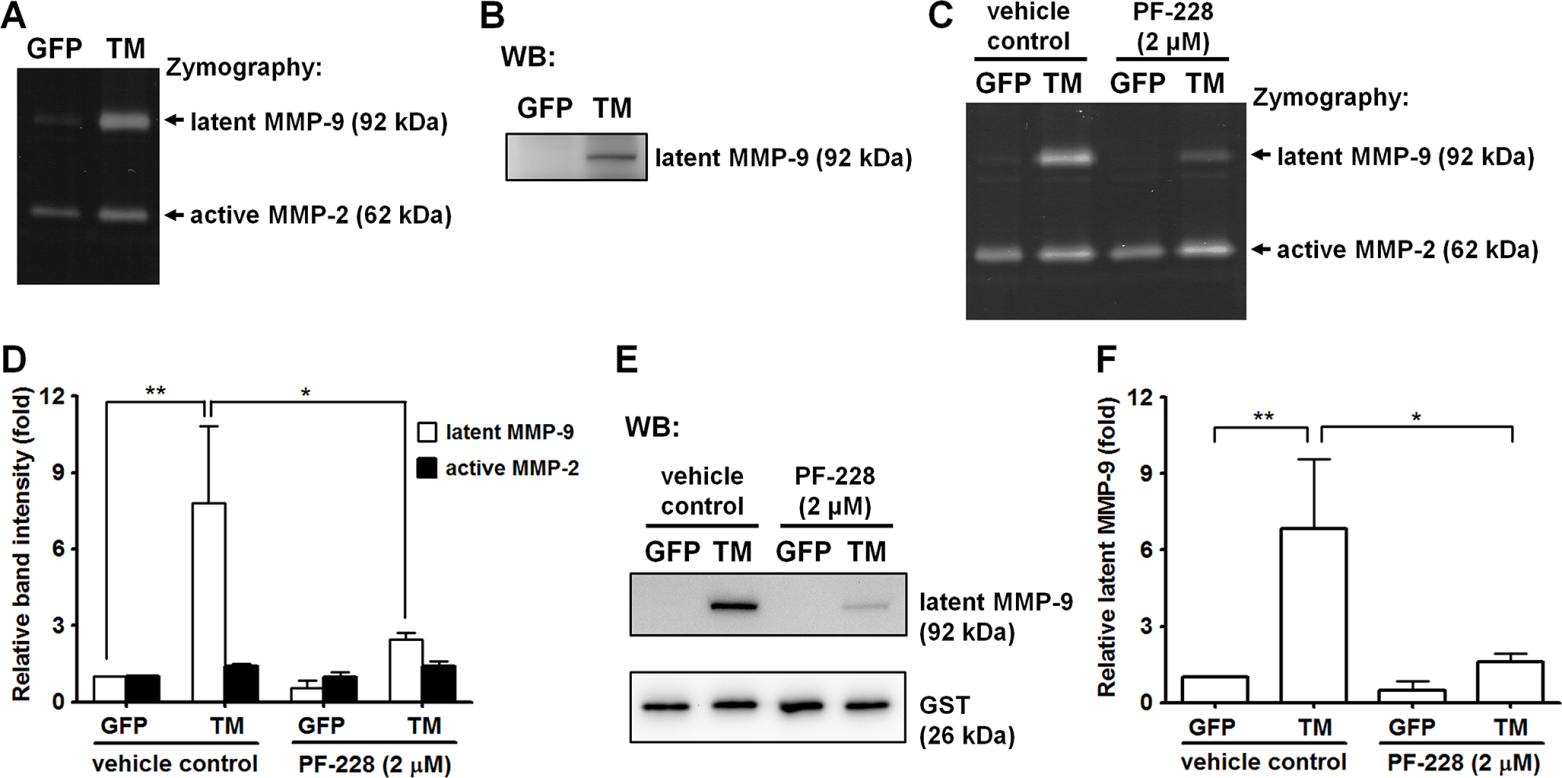
Exogenous expression of TM enhances MMP-9 production (**A**) MMP-9 and MMP-2 activities in the conditioned medium from GFP-expressing and GFP-tagged TM-expressing A2058 cells were assessed by gelatin zymography. (**B**) MMP-9 protein levels in the conditioned medium were analyzed by western blotting (WB). (**C**) GFP-expressing and GFP-tagged TM-expressing A2058 cells were grown in culture medium containing the FAK inhibitor PF-228 (2 μM) or vehicle control (DMSO) for 24 h. MMP-9 and MMP-2 activities in the conditioned medium with PF-228 or vehicle control were analyzed by gelatin zymography. (**D**) Quantification of band intensity of gelatin zymography. Values represent means ± SD of 3 independent experiments. (**E**) MMP-9 protein levels in the conditioned medium were analyzed by western blotting. GST protein, which was added to the conditioned medium before concentration, was used as a concentrating and loading control. (**F**) Quantification of MMP-9 protein levels. Values are means ± SD of 3 independent experiments. *P < 0.05 and **P < 0.01.

Previous studies showed that fibronectin stimulates MMP-9 expression via α5β1 integrin through activation of FAK/phosphatidylinositol 3-kinase (PI3K)/extracellular signal-regulated kinase (ERK) signaling [18]. Here, we investigated whether TM, a novel cell surface receptor for fibronectin, regulates MMP-9 expression. A2058 cells expressing GFP or GFP-tagged TM were grown in culture medium for 24 h and maintained in serum-free medium for another 24 h. The conditioned medium was analyzed by gelatin zymography and western blotting. In gelatin zymography assays, gelatinases (MMP-2 and MMP-9) are denatured in the presence of SDS, exposing their active sites. Both the latent and active forms of gelatinases exhibit gelatinolytic activities after the partial renaturation [19]. Our gelatin zymography assay showed that the gelatinolytic activity of latent MMP-9 (pro-MMP-9) was higher in GFP-tagged TM-expressing A2058 cells than in GFP-expressing cells (Figure 5A). Western blot analysis demonstrated that protein levels of latent MMP-9 were higher in GFP-tagged TM-expressing A2058 cells than in GFP-expressing cells (Figure 5B). To examine whether FAK signaling is required for TM-mediated MMP-9 expression, we treated cells with the FAK inhibitor PF-228 or vehicle control DMSO before conditioned medium collection. Gelatin zymography and western blotting showed that PF-228 treatment significantly inhibited latent MMP-9 protein levels in GFP-tagged TM-expressing A2058 cells (Figure 5C–5F), suggesting that TM triggers MMP-9 expression by activating FAK. To investigate whether the cytoplasmic domain of TM modulates the expression of MMP-9, we examined the MMP-9 protein level of A2058 cells expressing cytoplasmic domain-deleted TM (TM(ΔC)GFP). As shown in Supplementary Figure S2, the MMP-9 protein level of the conditioned medium was decreased in A2058 cells expressing cytoplasmic domain-deleted TM compared with that in A2058 cells expressing GFP-tagged TM. This result implies that TM stimulates MMP-9 expression through its cytoplasmic domain.

### Endogenous TM promotes endothelial cell adhesion and FAK tyrosine phosphorylation

**Figure 6:**
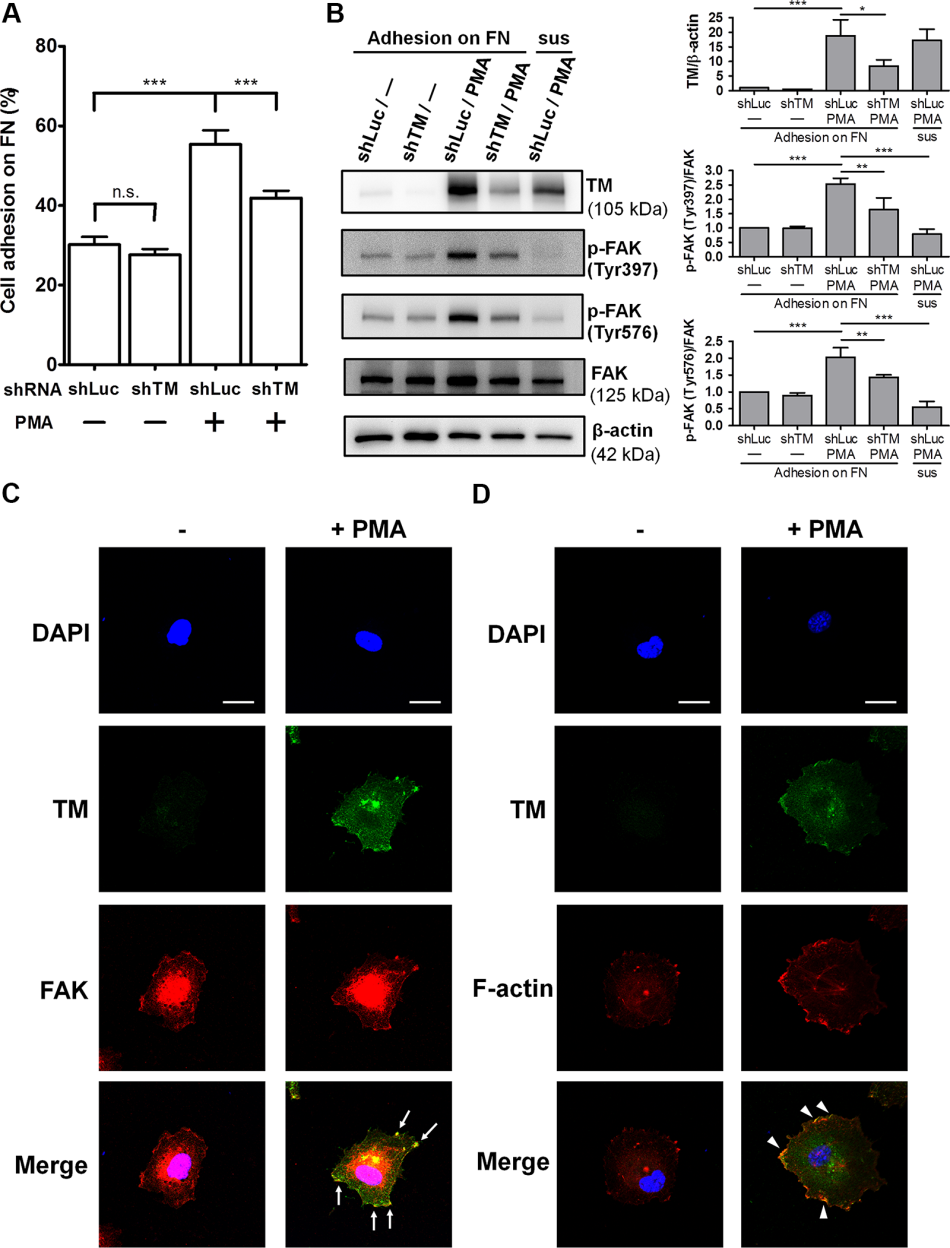
Endogenous TM promotes endothelial cell adhesion and FAK tyrosine phosphorylation (**A**) HUVECs were infected with lentiviruses expressing control luciferase shRNA (shLuc) or TM shRNA (shTM) for 24 h. These cells were treated without (−) or with (+) 10 nM PMA for 48 h. The cells were loaded onto fibronectin-coated wells and incubated for 45 min. After washing twice, endogenous phosphatase activities of adherent cells were measured. Data are expressed as the percentage of adherent cells to total cells loaded. Values represent means ± SD of quadruplicate wells. (**B**) HUVECs, which were infected with shLuc or shTM lentiviruses, were treated without (−) or with 10 nM PMA for 48 h. The cells were suspended (sus) or plated on fibronectin-coated dishes for 1 h. Cell lysates were analyzed by western blotting. Right panels show quantitative results of TM levels normalized by β-actin and that of phospho-FAK levels normalized by FAK. Values are means ± SD of 3 independent experiments. (**C**) Untreated or PMA-treated HUVECs were plated on fibronectin-coated coverslips for 1 h, then doubly stained with anti-TM and anti-FAK antibodies, and analyzed by confocal fluorescence microscopy. Arrows indicate co-localization of TM and FAK at the cell spreading edges. (**D**) Untreated or PMA-treated HUVECs plated on fibronectin for 1 h were doubly stained with an anti-TM antibody and Alexa Fluor 555 phalloidin. Arrowheads point to co-localization of TM and F-actin at the cell spreading edges. Several fields of the immunofluorescence staining were observed. Scale bars = 20 μm. Results are representative of 3 independent experiments. *P < 0.05, **P < 0.01, and ***P < 0.001. n.s., not significant.

Studies showed that TM expression in endothelial cells is up-regulated by several stimulators, including phorbol 12-myristate 13-acetate (PMA) [20], histamine [21], retinoic acid [22], and vascular endothelial growth factor (VEGF) [23], through increasing TM transcription. To investigate whether TM mediates endothelial cell-fibronectin adhesion, we used PMA to stimulate TM expression in human umbilical vein endothelial cells (HUVECs) and performed cell adhesion assays. Additionally, we utilized short hairpin RNA (shRNA) to knock down TM expression to verify the role of TM in endothelial cell adhesion. HUVECs were infected with lentiviruses expressing control luciferase shRNA (shLuc) or TM shRNA (shTM). These cells were treated with 10 nM PMA for 48 h, and then subjected to cell adhesion assays. In shLuc-expressing cells, PMA treatment increased cell adhesion to fibronectin by 1.8-fold, whereas TM knockdown in PMA-treated cells decreased cell adhesion compared with shLuc-expressing PMA-treated cells (Figure 6A). Next, we assessed whether TM-mediated cell adhesion in PMA-treated cells is dependent on integrins. Fibronectin-binding integrins recognize the RGD sequence of fibronectin, and RGD peptides have been used to block integrin functions [24, 25]. We used the Gly-Arg-Gly-Asp-Ser-Pro (GRGDSP) peptide, which is a RGD-based integrin-blocking peptide, to block integrin binding to fibronectin. The cell adhesion assay showed that the GRGDSP peptide suppressed the adhesion of PMA-untreated and PMA-treated HUVECs to fibronectin (Supplementary Figure S3). However, PMA treatment still promoted a 1.68-fold increase in cell adhesion even in the presence of the GRGDSP peptide (Supplementary Figure S3, bars 2 and 4). This result suggests that the GRGDSP peptide inhibits integrin-mediated cell adhesion, but does not abolish TM-mediated cell adhesion.

To explore the effect of endothelial TM on FAK phosphorylation, we used PMA and shTM to modulate TM expression levels in HUVECs and then analyzed FAK phosphorylation following cell adhesion on fibronectin. HUVECs, which were infected with shLuc or shTM lentiviruses, were untreated or treated with 10 nM PMA for 48 h. These cells were plated on fibronectin and incubated for 1 h. In shLuc-expressing cells, PMA treatment effectively induced TM overexpression and markedly increased FAK phosphorylation on Tyr397 and Tyr576 (Figure 6B). Compared with shLuc-expressing PMA-treated cells, shTM-expressing PMA-treated cells, in which TM expression was knocked down with an efficiency close to 60%, exhibited an obvious decrease in FAK phosphorylation on Tyr397 and Tyr576 (Figure 6B). In contrast, when shLuc-expressing PMA-treated cells were kept in suspension for 1 h, FAK phosphorylation levels on Tyr397 and Tyr576 were low. Taken together, these data suggest that TM increases endothelial cell-fibronectin adhesion and promotes FAK tyrosine phosphorylation in endothelial cells.

### TM co-localizes with FAK and actin filaments at the cell spreading edges

We next examined the distribution of TM and FAK in endothelial cells spread on fibronectin. HUVECs were treated with PMA for 48 h to up-regulate TM expression. These cells were harvested and plated on fibronectin for 1 h, followed by immunofluorescence staining. Confocal microscopy images showed that staining for TM was weak in PMA-untreated cells and TM expression was induced by PMA (Figure 6C). In PMA-treated cells, TM was concentrated at the edges of the membrane extensions where it co-localized with FAK (Figure 6C, arrows). We recently found that TM indirectly associates with the actin cytoskeleton via the actin-binding protein ezrin in epithelial cells [13]. We identified ^522^RKK^524^ of the TM cytoplasmic domain as important ezrin-binding residues [13]. Therefore, we examined whether TM is linked to actin filaments in endothelial cells. Immunofluorescence staining revealed that, in PMA-treated HUVECs, TM co-localized with actin filaments at the spreading edges (Figure 6D, arrowheads). These results indicate that endothelial TM is located to the cell periphery together with FAK and actin filaments during cell spreading.

### TM enhances endothelial tube formation *in vitro*

**Figure 7:**
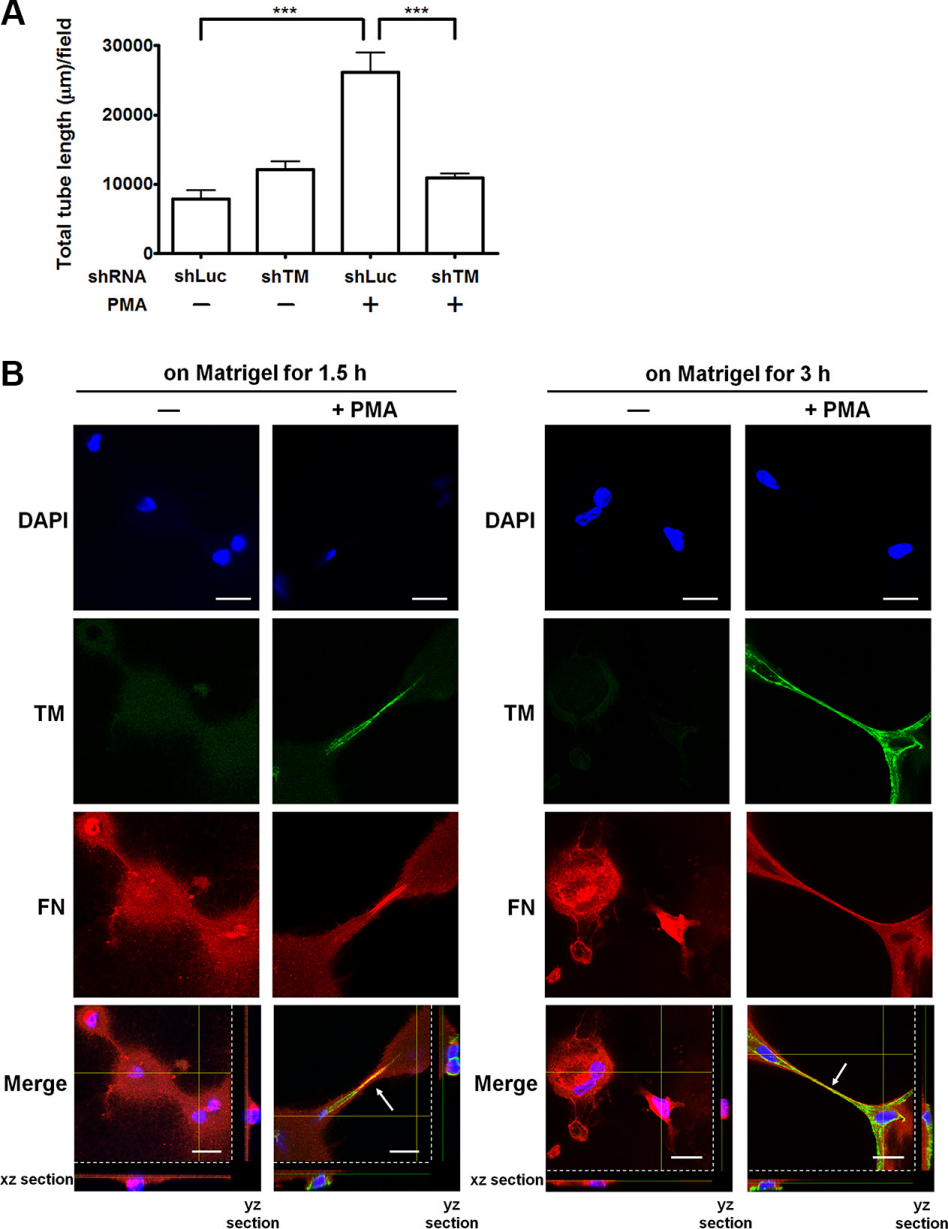
TM enhances endothelial tube formation and fibronectin fibrillogenesis *in vitro* (**A**) Effects of TM up-regulation and TM knockdown on tube formation. HUVECs were infected with shLuc or shTM lentiviruses. These cells were treated without (−) or with (+) 10 nM PMA in M199 containing 5% FBS for 48 h and then seeded on Matrigel in M199 containing 5% FBS. Tube structures were photographed after 5 h of incubation. Graph shows quantitative analysis of the total tube length per field. Values are means ± SD (*n* = 3 separate wells), and similar results were obtained in 3 independent experiments. ***P < 0.001. (**B**) The distribution of TM and fibronectin on HUVECs during tube formation. HUVECs were untreated (−) or treated with (+) 10 nM PMA in M199 containing 5% FBS for 48 h. The cells were plated on Matrigel-coated coverslips in M199 containing 5% FBS for 1.5 or 3 h. Immunofluorescence staining was performed with anti-TM and anti-fibronectin antibodies and analyzed by confocal microscopy. Arrows indicate co-localization of TM and fibronectin at the extending tube-like structures. The xz and yz sections show the location of the nuclei. Scale bars = 20 μm. Data are representative of 3 independent experiments.

Fibronectin is an important matrix associated with angiogenesis [16]. We hypothesized that endothelial TM functions as one of fibronectin receptors to promote angiogenesis. To test this hypothesis, we examined whether overexpression of TM enhances endothelial tube formation *in vitro* and whether TM knockdown impairs the tube formation. HUVECs, which were infected with shLuc or shTM lentiviruses, were untreated or treated with PMA (10 nM) for 48 h and then subjected to the tube formation assay. In this assay, the cells were suspended in M199 with 5% FBS, which contains plasma fibronectin, and added to the μ-slide well coated with Matrigel. Control shLuc-expressing cells formed a small amount of tubes, while the shLuc-expressing cells, when stimulated with PMA to induce TM expression, developed elongated tube-like structures (Figure 7A and Supplementary Figure S4). In contrast, TM knockdown in PMA-treated cells significantly reduced the tube formation compared with shLuc-expressing PMA-treated cells (Figure 7A and Supplementary Figure S4).

Next, we examined the distribution of TM and fibronectin during *in vitro* endothelial tube formation. Untreated or PMA-treated HUVECs were plated on Matrigel-coated coverslips. After 1.5 h or 3 h of incubation, immunofluorescence staining was performed. Compared with untreated cells, PMA-treated cells displayed strong TM staining and obvious tube formation on Matrigel at both time points (Figure 7B). TM and fibronectin co-localized at the tube-like structures of PMA-treated cells (Figure 7B, arrows). These results indicate that TM is involved in endothelial tube formation and co-localizes with fibronectin at the tube-like structures. This observation implies that TM interacts with fibronectin to extend and maintain the endothelial tube structures.

### TM is expressed on tumor blood vessels and binds to fibronectin during tumor angiogenesis

**Figure 8:**
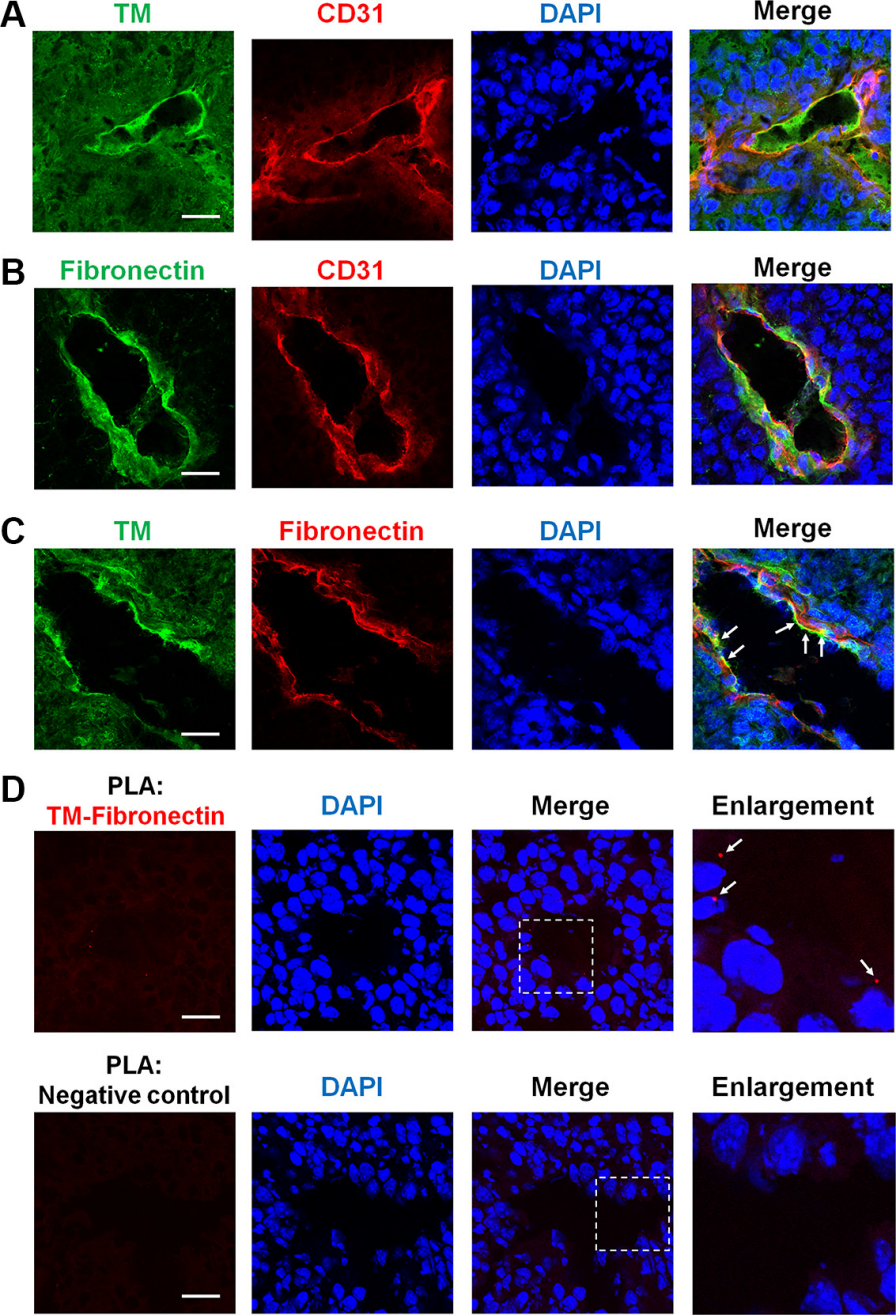
TM is expressed on tumor blood vessels and binds to fibronectin during tumor angiogenesis B16F10 melanoma cells (1 × 10^5^) were injected subcutaneously into C57BL/6 mice. After 17 days, tumors were harvested, and tumor sections were processed for immunofluorescence staining with specific antibodies. (**A**) Representative immunofluorescence staining of TM and CD31 (an endothelial marker) in tumors. TM is expressed on the tumor endothelium. (**B**) Representative immunofluorescence staining of fibronectin and CD31 in tumors. Fibronectin is also detected on the tumor endothelium. (**C**) Representative immunofluorescence staining of TM and fibronectin in tumors. Arrows indicate co-localization of TM and fibronectin on the tumor blood vessels. (**D**) Visualization of TM-fibronectin interactions in tumor sections by the proximity ligation assay (PLA). Tumor sections were fixed and subjected to the PLA using anti-TM and anti-fibronectin primary antibodies. TM-fibronectin interactions were detected by confocal microscopy as red fluorescent spots. Nuclei were stained with DAPI (blue). Arrows in the enlarged image point to TM-fibronectin interactions on the tumor blood vessel. A section processed without primary antibodies was used as a negative control. No PLA signal was observed in the negative control. Scale bars = 30 μm.

Angiogenesis that occurs in tumors may lead to further tumor progression and metastasis [2]. Here, we addressed whether TM is involved in tumor angiogenesis. We performed immunofluorescence staining on frozen biopsies of melanoma tumors, which were derived from C57BL/6 mice subcutaneously injected with B16F10 murine melanoma cells. Transplantation of B16F10 cells in mice for 17 days gave rise to tumors harboring a number of blood vessels as judged by staining of CD31, a vascular endothelial cell marker. Confocal microscopy images revealed that TM and fibronectin were present on CD31-positive blood vessels in the tumors (Figure 8A and 8B). In a double staining analysis of TM and fibronectin, we found that TM co-localized with fibronectin on the tumor blood vessels (Figure 8C, arrows). The proximity ligation assay (PLA) is a technique that monitors endogenous protein-protein interactions *in situ* [26]. To further confirm the TM-fibronectin interaction during tumor angiogenesis, we performed the PLA using Duolink *In Situ* reagents. Tumor sections were stained with anti-TM and anti-fibronectin primary antibodies followed by the PLA. TM-fibronectin interactions were visualized as red fluorescent spots that were present on the tumor blood vessel (Figure 8D, arrows in the enlarged image). No red spot was detected when the primary antibodies were omitted (Figure 8D, negative control). These results indicate that TM is expressed on the tumor vasculature and interacts with fibronectin during tumor angiogenesis.

### TM knockdown attenuates VEGF-induced endothelial cell adhesion and tube formation

**Figure 9:**
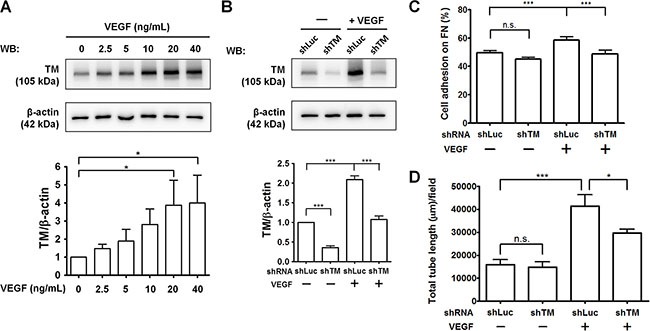
TM knockdown attenuates VEGF-induced endothelial cell adhesion and tube formation (**A**) HUVECs were starved in M199 (5% FBS) for 24 h and treated with various concentrations of VEGF for another 24 h. TM expression was analyzed by western blotting (WB) and quantified. The bottom panel shows quantitative results of TM levels normalized by β-actin, and values represent means ± SD of 3 independent experiments. (**B**–**D**) HUVECs, which were infected with shLuc or shTM lentiviruses, were starved in M199 (5% FBS) for 24 h and treated without (−) or with (+) 20 ng/mL VEGF for another 24 h. B. TM expression was analyzed by western blotting. The bottom panel shows quantitative results of TM levels normalized by β-actin. Values are means ± SD of 3 independent experiments. C. The cells were plated on fibronectin-coated wells. After 45 min of incubation, endogenous phosphatase activities of adherent cells were measured. Data are expressed as the percentage of adherent cells to total cells loaded. Values represent means ± SD of quadruplicate wells. D. The cells were plated on Matrigel in M199 containing 5% FBS. After 5 h, tube structures were photographed. The total tube length per field was measured. Values represent means ± SD of triplicate wells. Data are representative of 3 independent experiments. *P < 0.05 and ***P < 0.001. n.s., not significant.

Tumor cells and tumor-associated macrophages can secrete VEGF, which is a potent inducer of tumor angiogenesis [2]. To investigate the potential role of TM in VEGF-induced angiogenesis, we examined TM protein levels in HUVECs in response to VEGF. As shown in Figure 9A, VEGF dose-dependently induced TM expression in HUVECs. VEGF increased TM levels by 3.8-fold at a concentration of 20 ng/mL. We have previously demonstrated that platelet-derived growth factor-BB (PDGF-BB) stimulates TM expression in vascular smooth muscle cells and murine corneal epithelial cells via the mammalian target of rapamycin (mTOR) pathway [27, 28]. Therefore, we examined whether VEGF induces TM expression in an mTOR-dependent manner. Pre-treatment of the mTOR inhibitor rapamycin abolished VEGF-stimulated TM expression in HUVECs (Supplementary Figure S5). Subsequently, we examined the effects of TM knockdown on VEGF-dependent endothelial cell adhesion and tube formation. HUVECs were infected with shLuc or shTM lentiviruses, starved in M199 (5% FBS) for 24 h, and treated with or without 20 ng/mL VEGF for another 24 h. Western blotting showed that the VEGF-induced TM expression was knocked down to the basal level by shTM (about 50% inhibition) (Figure 9B). In cell adhesion experiments, treatment of shLuc-expressing cells with VEGF promoted cell adhesion to fibronectin, whereas TM knockdown in VEGF-treated cells significantly decreased cell adhesion (Figure 9C). In addition, VEGF treatment markedly induced tube formation of shLuc-expressing cells on Matrigel by 2.6-fold, while TM knockdown reduced VEGF-stimulated tube formation (Figure 9D and Supplementary Figure S6). These results indicate that TM mediates VEGF-induced endothelial cell adhesion and tube formation, implying that TM has a role in VEGF-dependent tumor angiogenesis.

## DISCUSSION

Angiogenesis is a complex process, involving cell-matrix adhesion, cell migration, ECM remodeling, and tube formation. Understanding the molecular and cellular basis of angiogenesis may help develop pro- or anti-angiogenesis treatments for diverse diseases. The expression of TM is up-regulated by angiogenic factors, but the role of TM in angiogenesis remains mostly unknown. In this study, we demonstrated that TM is a cell-matrix adhesion molecule contributing to angiogenesis. We first identified that rTMD1 directly interacts with fibronectin and, to a lesser extent, collagen I (Figure 1B). Moreover, the anti-TM antibody could block rTMD1 binding to fibronectin (Figure 1C), confirming that the interaction between rTMD1 and fibronectin is specific. Similarly, Tomkowicz *et al.* showed that endosialin, which presents some homology with TM in the N-terminal extracellular domain, binds to fibronectin and collagen I through its lectin-like domain [29]. Surprisingly, although TM and endosialin interacted with collagen I, both proteins did not bind to gelatin (denatured collagen) in our study and in the previous report [29].

Fibronectin is a large glycoprotein that can bind to other ECM proteins, integrins, growth factors, and syndecans via its diverse domains [30]. The RGD motif within the fibronectin type III10 module is known to bind to α5β1 integrin [16]. By testing different fragments of fibronectin, we demonstrated that the N-terminal 70-kDa domain of fibronectin was the rTMD1-binding site (Figure 2B). In the solid-phase binding assay, full-length fibronectin (10 μg/mL) and its fragments (equimolar) were coated onto wells. The interaction of the 70-kDa fragment of fibronectin with rTMD1 is almost half that of full-length fibronectin (Figure 2B). A possible explanation is that full-length fibronectin, which is a bigger protein of about 230 kDa, may expose more TM-binding sites than its N-terminal 70-kDa fragment after being coated onto the well surface. rTMD1 can bind to the 30-kDa and 45-kDa fragments of fibronectin (Figure 2B), therefore the interactions may be additive. It is possible that fibronectin has the ability to bind to TM and α5β1 integrin simultaneously via its distinct molecular surfaces. Integrins are known to interact with ECM proteins to regulate cell adhesion, intracellular signaling, and migration [3]. Since the TM lectin-like domain had the ability to interact with fibronectin, we examined the effects of TM overexpression on cell adhesion, intracellular signaling, and migration. Our data showed that exogenous expression of TM in TM-deficient A2058 cells promoted cell adhesion, FAK tyrosine phosphorylation, and migration on fibronectin (Figures 3 and 4). The increased cell adhesion might result in increased cell migration. These findings suggest that TM, like integrins, acts as a cell-matrix adhesion molecule.

Angiogenesis requires proteolysis of the ECM that allows endothelial cell invasion, and MMP-9 has been demonstrated to play a key role in angiogenesis [31]. Following the interaction with ECM components, cell surface receptors may regulate gene expression by activating intracellular signaling pathways [3, 4]. Previous reports indicated that fibronectin, possibly via α5β1 integrin, stimulates the production of MMP-9 in human laryngeal carcinoma cells [18], breast cancer cells [32], and T lymphocyte cell lines [33]. In this study, we demonstrated that TM enhanced MMP-9 expression in A2058 cells (Figure 5A and 5B). This finding suggests that TM, similar to α5β1 integrin, can up-regulate MMP-9 expression in response to fibronectin. FAK has been implicated in regulating MMP-9 expression. Activation of FAK and its downstream PI3K/ERK signaling leads to MMP-9 expression upon fibronectin binding to integrins [18, 34]. In addition, hyaluronic acid, which is a glycosaminoglycan that binds to cell surface CD44, also induces MMP-9 expression in glioma cells via activation of the FAK signaling pathway [35]. In our study, inhibition of FAK activation by PF-228 significantly diminished MMP-9 expression in TM-expressing A2058 cells (Figure 5C−5F). These data suggest that FAK activation is required for TM-mediated up-regulation of MMP-9, consistent with previous studies showing the positive effect of FAK on MMP-9 production.

Recently, we demonstrated that human plasminogen kringle 1–5 binds to TM on endothelial cells and induces TM internalization and degradation, thereby inhibiting angiogenesis [36]. However, the mechanisms by which TM regulates angiogenesis remain largely unclear. Here, we found that up-regulation of TM by PMA in HUVECs promoted cell adhesion on fibronectin and enhanced FAK phosphorylation on Tyr397 and Tyr576, whereas TM knockdown inhibited these events (Figure 6A and 6B). Knockdown of the basal expression of TM did not affect the adhesion of HUVECs to fibronectin (Figure 6A, bar 2). One possible explanation is that the low levels of TM did not influence cell adhesion to fibronectin (Figure 6A, bars 1 and 2). Previous studies showed that PMA activates protein kinase C (PKC) to facilitate epithelial cell migration, and that activation of ERK by PKCε is essential for PMA-dependent adhesion and migration in human glioma cells [37, 38]. However, the long-term effects of PMA on cell-matrix interactions remain undefined. We demonstrated here that PMA induces expression of TM, which can bind to fibronectin to increase cell adhesion and FAK activation. Furthermore, TM co-localizes with FAK and actin filaments at the spreading edge of PMA-treated HUVECs (Figure 6C and 6D). FAK has been suggested to play an important role in angiogenesis. Knockout of endothelial FAK affects mouse embryogenesis with impaired angiogenesis [39]. In contrast, FAK overexpression in transgenic mice promotes angiogenesis in wound healing and hindlimb ischemia models [40]. Therefore, TM may promote angiogenesis by enhancing call adhesion and FAK activation via interacting with fibronectin. It cannot be excluded that an increase in adhesion by TM induces greater integrin signaling to FAK.

We further demonstrated that up-regulation of TM expression by PMA in HUVECs promotes tube formation on Matrigel, and TM knockdown suppresses tube formation (Figure 7A). These results are consistent with our previous report showing that TM degradation impairs endothelial tube formation [36]. Fibronectin assembly (also termed fibronectin fibrillogenesis) is initiated by binding of fibronectin to cell surface receptors, such as α5β1 integrin [17]. Fibronectin binding to the cell surface leads to its conformational changes to unmask cryptic sites involved in fibronectin self-assembly. Fibronectin assembly on endothelial cells is required for neovessel formation [41]. During *in vitro* endothelial tube formation, TM co-localized with fibronectin at tube-like structures (Figure 7B), suggesting that TM functions as a fibronectin receptor that promotes fibronectin assembly in angiogenesis. Besides TM, the TM-related family member CD93 has been reported to be a regulator of angiogenesis [42]. The anti-CD93 monoclonal antibody has been shown to partially impair angiogenesis in mice [43].

Angiogenesis can occur in physiological and pathological situations [2]. In solid tumors, new blood vessels, which provide oxygen and nutrients for tumor cells and remove waste products, promote tumor growth and metastasis [44]. Interactions of endothelial cell integrins with the ECM have been suggested to contribute to tumor angiogenesis [2]. Here, we found that both TM and fibronectin were detected on CD31-positive tumor vascular endothelium of mouse melanomas (Figure 8A and 8B). TM co-localized with fibronectin on the tumor blood vessels (Figure 8C, arrows), implying that fibronectin molecules bind to endothelial TM during tumor angiogenesis. We observed that fibronectin was partially, but not fully, co-localized with TM, meaning that some fibronectin molecules may bind to other cell surface receptors like integrins. In addition, we used the PLA to show the TM-fibronectin interactions on the tumor blood vessels (Figure 8D, arrows in the enlarged area). Consistently, it was reported that fibronectin matrix assembly is dramatically increased in the neovessels of human renal cell carcinomas and breast carcinomas, compared with that observed in normal tissues [41].

VEGF plays a critical role in tumor angiogenesis by inducing sprouting and tube formation of endothelial cells [2]. The anti-VEGF monoclonal antibody (Avastin) has been approved for treating patients with colorectal cancer [45]. An early report showed that VEGF increases cell surface TM expression in human aortic endothelial cells [23]. In accordance with this report, we found that TM expression was up-regulated by VEGF in HUVECs (Figure 9A). We hypothesized that VEGF induces TM levels to promote endothelial cell adhesion and tube formation. Consistent with our hypothesis, TM knockdown impaired VEGF-induced HUVEC adhesion on fibronectin and tube formation (Figure 9C and 9D). Thus, in tumors, VEGF stimulates TM expression in endothelial cells where TM may act as a fibronectin receptor, contributing to tumor angiogenesis. Our previous report showed that PDGF-BB stimulated TM expression by the Src kinase/PI3K/Akt/mTOR pathway [27]. In the present study, we found that VEGF-induced TM expression was attenuated by the mTOR inhibitor rapamycin (Supplementary Figure S5). Rapamycin has been shown to inhibit tumor growth and angiogenesis in mice [46]. The anti-angiogenic effect of rapamycin might be in part attributed to its ability to inhibit TM expression. To date, integrin antagonists, including specific antibodies and peptides, have been evaluated in clinical trials for cancer therapy [2]. Targeting TM by its antagonists, such as TM-specific antibodies or plasminogen kringle domains, may be useful to inhibit tumor angiogenesis and growth.

In conclusion, our results indicate that TM promotes angiogenesis by enhancing cell adhesion, migration, FAK activation, and fibronectin assembly via interacting with the ECM fibronectin. This study suggests that tumor endothelial TM is associated with tumor angiogenesis and may represent a novel anti-angiogenic target. Future studies should aim at developing TM antagonists to evaluate its potential for anti-angiogenic therapy.

## MATERIALS AND METHODS

### Reagents

Human placenta collagen type I (Sigma C7774) and IV (Sigma C5533), laminin (Sigma L6274), human plasma fibronectin proteolytic fragments 70 kDa (Sigma F0287), 30 kDa (Sigma F9911), 45 kDa (Sigma F0162), BSA, PMA, GRGDSP peptide, and the FAK inhibitor PF-228 were purchased from Sigma-Aldrich (St. Louis, MO, USA). Human plasma fibronectin and its 120-kDa α-chymotryptic fragment were obtained from Millipore (Temecula, CA, USA). Human plasma vitronectin (catalog no. G5381) was purchased from Promega (Madison, WI, USA). Recombinant fibronectin fragment 2, fragment 4, and VEGF were purchased from R&D Systems (Minneapolis, MN, USA). Gelatin was from Merck (Darmstadt, Germany). Growth factor reduced Matrigel was obtained from Corning (Bedford, MA, USA).

### Preparation of rTMD1 protein

The vector pCR3 (Invitrogen, Carlsbad, CA, USA) expressing rTMD1 with a c-Myc epitope and 6× His tag was constructed and transfected into HEK293 cells previously [15]. Cell medium containing expressed rTMD1 was purified by a nickel-chelating Sepharose column (GE healthcare, Piscataway, NJ, USA). Purified rTMD1 was analyzed by SDS-PAGE and western blotting using an anti-His antibody (AbD Serotec, Oxford, UK).

### Solid-phase binding assay

Maxisorp plate (Nunc, Roskilde, Denmark) wells were coated with 10 μg/mL fibronectin, vitronectin, collagen type I, collagen type IV, laminin or gelatin in carbonate buffer (pH 9.6) overnight at 4°C. The wells were blocked with 1% BSA in PBS for 2 h at room temperature. Various concentrations of rTMD1 diluted in PBS containing 0.1% BSA were added to wells and incubated overnight at 4°C. After washing with PBS containing 0.05% Tween 20 (PBST), the anti-His antibody was added to wells and incubated for 1.5 h at 37°C, followed by incubation with horseradish peroxidase-conjugated secondary antibody for 1 h at 37°C. The plate was washed and then developed with tetramethylbenzidine substrate, and the reaction was stopped with 1 M H_2_SO_4_. The absorbance at 450 nm was measured.

In antibody inhibition experiments, rTMD1 was mixed with increasing concentrations of the anti-TM antibody (sc-13164; Santa Cruz Biotechnology, Santa Cruz, CA, USA) and incubated for 1 h at 4°C. rTMD1 with or without the anti-TM antibody was then added to fibronectin-coated wells and incubated overnight at 4°C. Nonimmune mouse IgG was used as a control. Bound rTMD1 was determined as described above. To characterize the binding site for rTMD1, full-length fibronectin (10 μg/mL) and its fragments (equimolar) in carbonate buffer were coated onto wells. After blocking with 1% BSA, rTMD1 was added to wells and incubated overnight at 4°C. Since recombinant fibronectin fragments 2 and 4 were modified by fusion with His tag, bound rTMD1 was determined using an anti-c-Myc antibody (sc-40).

### Cell culture

A2058 cells stably expressing GFP-tagged human TM or GFP were generated and cultured as described previously [11]. HUVECs were cultured in M199 (Invitrogen) containing 20% FBS (Sigma-Aldrich), endothelial cell growth supplement (Millipore), heparin, and antibiotics. To determine the effect of VEGF on TM expression, HUVECs were starved for 24 h in M199 containing 5% FBS, and treated with various concentrations of VEGF diluted in M199 containing 5% FBS for another 24 h.

### TM knockdown by lentivirus-based RNA interference

The pLKO.1-shTM plasmid, which expresses shRNA against human TM, was obtained from the National RNAi Core Facility (Academia Sinica, Taipei, Taiwan). The target sequence of TM shRNA was 5′-GCCGATGTCATTTCCTTGCTA-3′ (clone ID: TRCN0000053923). The pLKO.1-shLuc plasmid encoding luciferase shRNA was used as a negative control. Lentiviruses expressing TM or luciferase shRNA were generated as described previously [13]. HUVECs were infected with lentiviruses for 24 h and used for experiments.

### Western blotting

A2058 cells expressing GFP-tagged TM or GFP were harvested using non-enzymatic cell dissociation solution (Sigma-Aldrich) or Accutase (Millipore), suspended in serum-free medium, and plated on fibronectin-coated dishes for 1 h. Cells were lysed with lysis buffer (0.6% Triton X-100, 100 mM Tris-HCl, pH7.4, and 1 mM PMSF) containing phosphatase inhibitors (1 mM Na_3_VO_4_ and 5 mM NaF) and protease inhibitors (Sigma-Aldrich). Cell lysates were resolved by SDS-PAGE. Western blotting was performed with antibodies against TM (sc-13164), phospho-FAK (Tyr397) (Cell Signaling Technology, Danvers, MA, USA), phospho-FAK (Tyr576) (sc-16563-R), and FAK (Invitrogen). Band intensities were quantified by ImageJ software (National Institutes of Health, Bethesda, MD, USA). To determine the role of endothelial TM in FAK phosphorylation, HUVECs, which were infected with lentiviruses expressing shLuc or shTM, were treated with or without 10 nM PMA in M199 containing 10% FBS for 48 h, and plated on fibronectin-coated dishes for 1 h. Western blotting was performed as described above. For western blot analysis of MMP-9 in the conditioned medium, glutathione *S*-transferase (GST) protein, which was prepared as described previously [13], was used as a concentrating and loading control. In brief, GST protein (0.3 μg) was added to the conditioned medium before concentration by Amicon centrifugal filter devices. Western blotting was carried out using antibodies against MMP-9 (sc-393859) and GST (sc-138).

### Cell adhesion assay

Maxisorp plate wells were coated with 10 μg/mL BSA or fibronectin overnight at 4°C. A2058 cells expressing GFP-tagged TM or GFP were harvested with non-enzymatic cell dissociation solution and diluted to a concentration of 3 × 10^5^ cells/mL in serum-free medium. Cell suspension (100 μL) was added to BSA- or fibronectin-coated wells. After 1 h, wells were washed five times with PBS. The adherent cells were quantitated by measuring endogenous phosphatase activities that were determined by incubation with 100 μL of lysis/substrate solution (50 mM sodium acetate, pH 5.0, 1% Triton X-100, and 6 mg/mL 4-nitrophenyl phosphate disodium) for 90 min at 37°C, followed by adding 50 μL of 1 M NaOH. The absorbance at 405 nm was measured. Data were expressed as the percentage of adherent cells compared with total cells added.

To evaluate the role of TM in endothelial cell adhesion, HUVECs were infected with shLuc or shTM lentiviruses and then treated with or without 10 nM PMA in M199 containing 10% FBS for 48 h. The cells (2 × 10^4^) were added to fibronectin-coated wells. After incubation for 45 min and two washes, the percentage of adherent cells was determined. In some experiments, HUVECs were infected with shLuc or shTM lentiviruses, and subcultured (1:2) in complete medium containing puromycin (1 μg/mL) for 24 h. These cells were starved in M199 containing 5% FBS for 24 h, and then stimulated with or without 20 ng/mL VEGF diluted in M199 containing 5% FBS for 24 h. Cell adhesion assay was performed as described above. To assess the effect of the GRGDSP peptide on HUVEC adhesion after TM up-regulation by PMA, HUVECs were treated with or without 10 nM PMA in M199 containing 10% FBS for 48 h, then suspended, and incubated with or without the GRGDSP peptide (0.2 mM) for 15 min at 37°C. The cells were added to fibronectin-coated wells and incubated for 45 min. After two washes, the percentage of adherent cells was determined as described above.

### Cell migration and invasion assays

A cell migration assay was performed using a 48-well Boyden chamber (Neuro Probe, Gaithersburg, MD, USA) and a polycarbonate filter with 8-μm pores (GE Healthcare). Both the top and bottom surfaces of the filter were coated with 10 μg/mL fibronectin. For determining the optimal migration conditions, we first tested Boyden chamber migration assays using 5% or 10% FBS as a chemoattractant in the lower compartment of the chamber. The best concentration of FBS for our experiments was 5% FBS. Therefore, the lower compartment of the chamber was filled with medium containing 5% FBS as a chemoattractant. 2 × 10^4^ cells in 50 μL of serum-free medium were added to the upper compartment. After 4 h, the filter was stained with Liu's stain (Handsel Technologies, Inc., Taipei, Taiwan). Cells on the filter upper surface were removed with cotton swabs. The migrated cells on the lower surface were photographed in 5 randomly selected fields under a microscope (×100 magnification) and counted. A cell invasion assay was performed using a 24-well Transwell plate with 8-μm pore polycarbonate membrane inserts (Corning, NY, USA). The porous membranes were coated with 40 μL of 1 mg/mL Matrigel. 4 × 10^4^ cells in 100 μL of medium containing 1% FBS were loaded onto the Matrigel-coated membrane in the upper compartment. Medium containing 5% FBS was added to the lower compartment as a chemoattractant. After 20 h of incubation at 37°C, the membranes were stained with Liu's stain. Cells on the membrane upper surface were removed with cotton swabs. Invading cells on the lower surface were photographed in 3 randomly selected fields under a microscope (×100 magnification) and counted.

### Gelatin zymography

A2058 cells expressing GFP-tagged TM or GFP (2 × 10^6^ cells) were seeded into 10-cm dishes and allowed to grow for 24 h. The cells were incubated in serum-free medium for 24 h, and the conditioned medium was concentrated using an Amicon centrifugal filter device (Millipore). Equal amounts of protein from samples were subjected to SDS-10% polyacrylamide gel containing 1 mg/mL gelatin. After electrophoresis, the gel was washed with 2.5% Triton X-100 in washing buffer (50 mM Tris-HCl, pH 7.4, and 100 mM NaCl) twice for 30 min each at 4°C to remove SDS and briefly with washing buffer. The gel was incubated in reaction buffer (50 mM Tris-HCl, pH 7.4, 100 mM NaCl, 10 mM CaCl_2_, 1 μM ZnCl_2_, and 0.02% NaN_3_) for 24 h at 37°C and subsequently stained with Coomassie Blue. Gelatinolytic activities of matrix metalloproteinases were detected as transparent bands. Furthermore, A2058 cells expressing GFP-tagged TM or GFP were treated with the FAK inhibitor PF-228 (2 μM) or vehicle control (DMSO) in culture medium for 24 h and incubated in serum-free medium with PF-228 (2 μM) or vehicle control (DMSO) for another 24 h. The conditioned medium was processed for gelatin zymography.

### Immunofluorescence

HUVECs were treated with or without 10 nM PMA in M199 containing 5% FBS for 48 h. These cells were harvested using Accutase, suspended in serum-free M199, and plated on fibronectin-coated coverslips for 1 h. Cells were fixed in 3.7% formaldehyde and permeabilized with 0.2% Triton X-100. Cells were blocked with 1% BSA and incubated with anti-TM (Dako, Carpinteria, CA, USA) and anti-FAK (Invitrogen) antibodies for 1.5 h at room temperature, followed by incubation with Alexa Fluor 488- or 546-conjugated secondary antibodies (Invitrogen) for 1.5 h at room temperature. Alexa Fluor 555 phalloidin (Invitrogen) was used to stain filamentous actin. Images were taken with an Olympus Fluoview FV1000 confocal laser scanning microscope using a ×63 objective (Olympus, Tokyo, Japan) at identical exposure times (12.5 μs/pixel). For staining of TM and fibronectin during tube formation, HUVECs, which were treated with or without 10 nM PMA in M199 containing 5% FBS for 48 h, were harvested using Accutase, suspended in M199 containing 5% FBS, and plated on Matrigel-coated coverslips for 1.5 or 3 h. Cells were doubly stained with anti-TM (Dako) and anti-fibronectin (F3648, Sigma-Aldrich) antibodies.

### *In vitro* tube formation assay

Tube formation assays were performed using 15-well μ-slides (ibidi GmbH, Martinsried, Germany). HUVECs were infected with shLuc or shTM lentiviruses, and then treated with or without 10 nM PMA in M199 containing 5% FBS for 48 h. Cells were harvested by Accutase, suspended in M199 containing 5% FBS, and added at a density of 5000 cells per well to the μ-slide well coated with 10 μL of Matrigel. After 5 h, wells were photographed with an Olympus microscope (× 40 magnification). The total tube length per field was measured using ImageJ. To investigate the role of TM in VEGF-related angiogenesis, HUVECs were infected with shLuc or shTM lentiviruses and subcultured (1:2) in complete medium containing puromycin (1 μg/mL) for 24 h. These cells were starved in M199 containing 5% FBS for 24 h, and then stimulated with or without 20 ng/mL VEGF in M199 containing 5% FBS for 24 h. The tube formation assay was performed.

### Subcutaneous tumor mouse model and immunofluorescence

A cell suspension (100 μL) containing 1 × 10^5^ B16F10 melanoma cells in PBS was subcutaneously injected into the dorsal flanks of 8-week-old C57BL/6 mice. After 17 days, tumors were harvested, embedded in Shandon Cryomatrix (Richard Allan Scientific, Kalamazoo, MI, USA), and frozen. Frozen tumors were cut into 5-μm sections. Sections were fixed in ice-cold acetone for 5 min, blocked with 1% BSA, and stained with rat anti-mouse CD31 (553370; BD Biosciences, San Diego, CA, USA) and rabbit anti-mouse TM lectin-like domain or rabbit anti-fibronectin (F3648) antibodies. The sections were then incubated with Alexa Fluor 546 goat anti-rat IgG and Alexa Fluor 488 goat anti-rabbit IgG. For double staining of TM and fibronectin, tumor sections were incubated with rabbit anti-mouse TM lectin-like domain and goat anti-fibronectin (sc-6952) antibodies, followed by incubation with Alexa Fluor 488 donkey anti-rabbit IgG and Alexa Fluor 546 donkey anti-goat IgG. Images were photographed using an Olympus Fluoview FV1000 confocal microscope with a × 40 objective at a scan speed of 12.5 μs/pixel. Four mice were evaluated in the subcutaneous tumor mouse model. At least three tumor sections were assessed by the staining. Animal care conditions and experiments were approved by the Institutional Animal Care and Use Committee of the National Cheng Kung University (Tainan, Taiwan).

### PLA

PLA was performed using the Duolink *In Situ* Orange Starter Kit Goat/Rabbit (DUO92106, Sigma-Aldrich) according to the manufacturer's instructions. Briefly, tumor sections of B16F10 melanoma cells as above were fixed in ice-cold acetone for 5 min, washed with PBS, and blocked with 1% BSA in PBS for 2 h at room temperature. 1% BSA in PBS was used for diluting primary antibodies and PLA probes. The tumor sections were incubated with rabbit anti-mouse TM lectin-like domain and goat anti-fibronectin (sc-6952) primary antibodies for 90 min at room temperature. After washing with PBST, the sections were incubated with anti-rabbit MINUS and anti-goat PLUS PLA probes for 60 min at 37°C. The sections were washed in 1 × wash buffer A and then incubated with ligation solution for 30 min at 37°C. After washing with 1 × wash buffer A, the sections were incubated with amplification solution for 100 min at 37°C. The samples were washed in 1 × wash buffer B and in 0.01 × wash buffer B and then mounted with Duolink *In Situ* Mounting Medium with DAPI. Images were taken using an Olympus confocal microscope with a × 40 objective at a scan speed of 12.5 μs/pixel.

### Statistical analysis

Data are expressed as means ± standard deviation (SD). Statistical significance was analyzed using paired or unpaired *t* test or one-way analysis of variance with a Bonferroni's test. Values of *P* < 0.05 were considered statistically significant.

## SUPPLEMENTARY MATERIALS FIGURES


