# Supplementary material for: Thrombomodulin promotes focal adhesion kinase activation and contributes to angiogenesis by binding to fibronectin
Source: Oncotarget. 2016 Sep 2;7(42):68122–39. doi: 10.18632/oncotarget.11828 (PMC5356543; doi:10.18632/oncotarget.11828)
Supplement: Supplementary file 1 [file oncotarget-07-68122-s001.pdf]

Thrombomodulin promotes focal adhesion kinase activation and contributes to angiogenesis by binding to fibronectin

Supplementary Materials

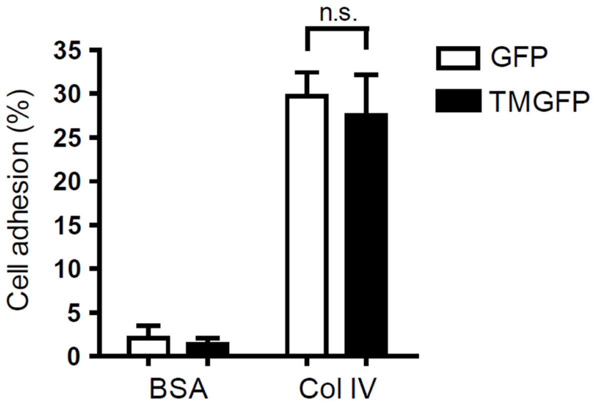

Supplementary Figure S1: Effect of exogenous TM expression on cell adhesion to collagen IV. A2058 cells expressing GFP or GFP-tagged TM (TMGFP) were seeded on wells coated with BSA (10 µg/mL) or collagen IV (Col IV, 10 µg/mL). After incubation for 1 h at 37°C and washing, adherent cells were quantitated by measuring endogenous phosphatase activities as described in Materials and Methods. Data were expressed as the percentage of adherent cells to total cells seeded. Values are means ± SD of quadruplicate wells. Data are representative of at least 3 independent experiments. n.s., not significant.

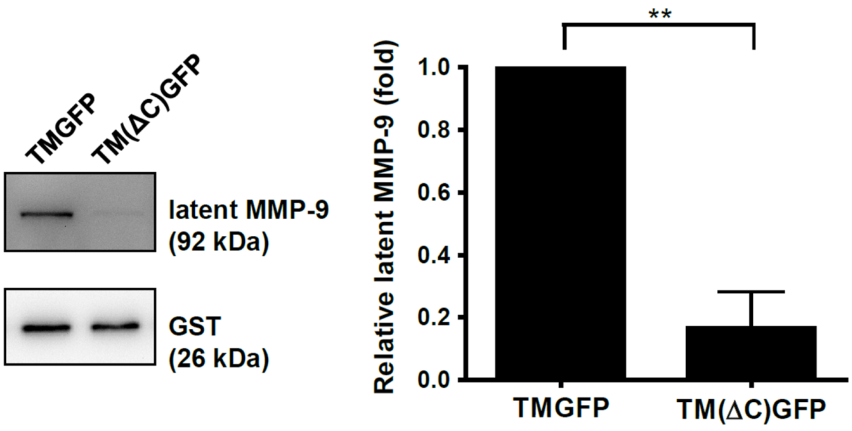

Supplementary Figure S2: Western blotting of serum-free conditioned medium of A2058 cells expressing GFP-tagged TM (TMGFP) or cytoplasmic domain-deleted TM (TM(ΔC)GFP). A2058 cells expressing TMGFP or TM(ΔC)GFP were seeded into 10-cm dishes and allowed to grow in culture medium for 24 h. The cells were incubated in serum-free medium for 24 h, and the conditioned medium was concentrated and subjected to western blot analyses. GST protein that was added to the conditioned medium before concentration was used as a control. The right panel shows quantitative results, and values represent means ± SD of 3 independent experiments. $^{**}P < 0.01$.

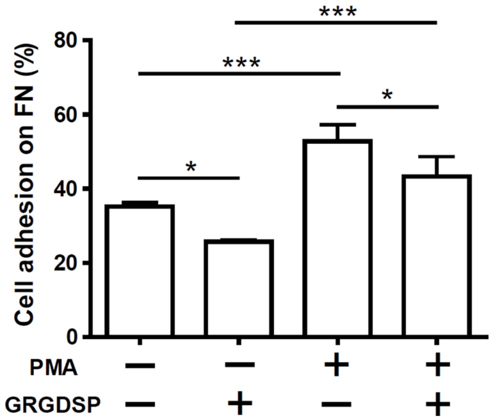

Supplementary Figure S3: Effect of the GRGDSP peptide on HUVEC adhesion to fibronectin. HUVECs were treated with or without 10 nM PMA in M199 containing 10% FBS for 48 h. These cells were suspended and incubated with the GRGDSP peptide (0.2 mM) for 15 min at 37°C. The cells were then seeded into fibronectin-coated wells and incubated for 45 min. After washing twice, endogenous phosphatase activities of adherent cells were measured. Results are expressed as the percentage of adherent cells to total cells seeded. Values are means ± SD of quadruplicate wells. Data are representative of 3 independent experiments. **P* < 0.05 and ****P* < 0.001.

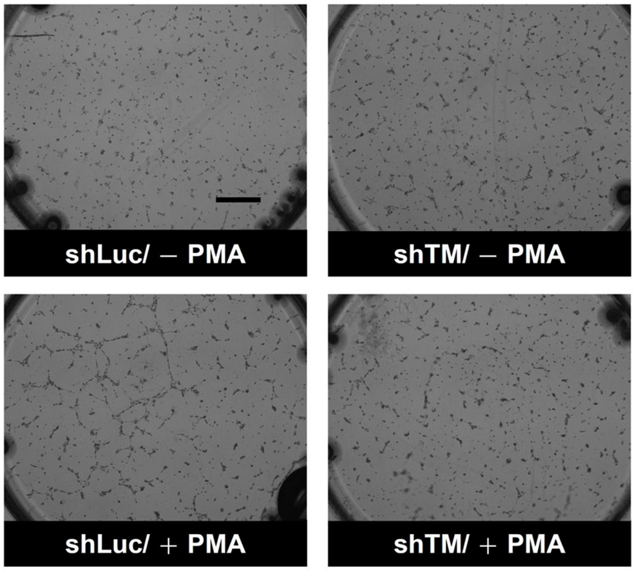

Supplementary Figure S4: Effect of TM knockdown on PMA-induced tube formation. HUVECs were infected with shLuc or shTM lentiviruses. These cells were treated without (−) or with (+) 10 nM PMA in M199 containing 5% FBS for 48 h and then seeded on Matrigel in M199 containing 5% FBS. Tube structures were photographed after 5 h of incubation. Scale bar = 0.5 mm.

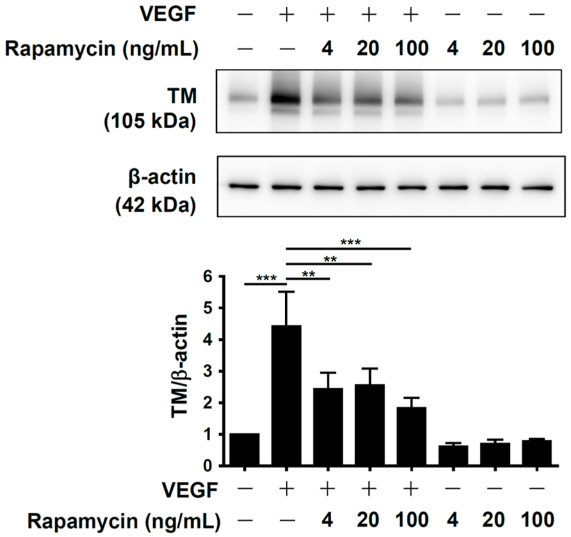

Supplementary Figure S5: Effect of rapamycin on VEGF-induced TM expression. HUVECs were starved in M199 containing 5% FBS for 24 h. These cells were treated with various concentrations of rapamycin, an inhibitor of mTOR, for 60 min and then stimulated with or without 20 ng/mL VEGF in M199 containing 5% FBS for 24 h. The expression levels of TM were analyzed by western blotting. β-Actin was used as a loading control. The bottom panel shows quantitative results, and values represent means ± SD of 3 independent experiments. $^{**}P < 0.01$ and $^{***}P < 0.001$.

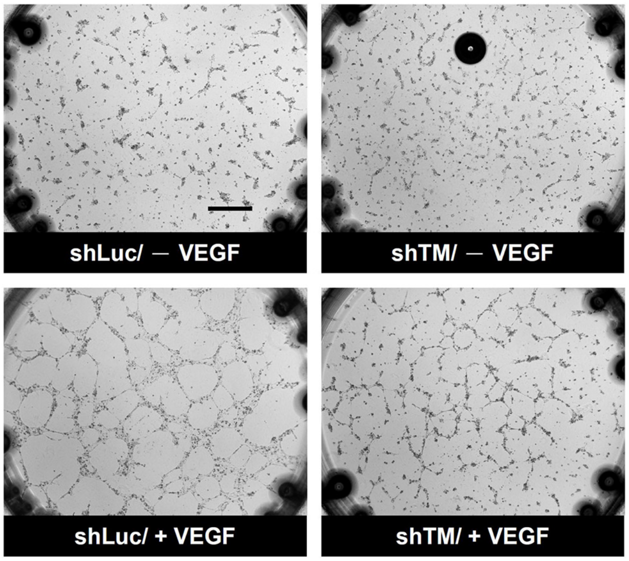

Supplementary Figure S6: Effect of TM knockdown on VEGF-induced tube formation. HUVECs, which were infected with shLuc or shTM lentiviruses, were starved in M199 containing 5% FBS for 24 h and treated without (−) or with (+) 20 ng/mL VEGF in M199 containing 5% FBS for another 24 h. The cells were seeded on Matrigel in M199 containing 5% FBS. After 5 h, tube structures were photographed. Scale bar = 0.5 mm.
